# Alzheimer disease research in the 21^st^ century: past and current failures, new perspectives and funding priorities

**DOI:** 10.18632/oncotarget.9175

**Published:** 2016-05-04

**Authors:** Francesca Pistollato, Elan L. Ohayon, Ann Lam, Gillian R. Langley, Thomas J. Novak, David Pamies, George Perry, Eugenia Trushina, Robin S.B. Williams, Alex E. Roher, Thomas Hartung, Stevan Harnad, Neal Barnard, Martha Clare Morris, Mei-Chun Lai, Ryan Merkley, P. Charukeshi Chandrasekera

**Affiliations:** ^1^ Physicians Committee for Responsible Medicine, Washington, DC, USA; ^2^ Green Neuroscience Laboratory, Neurolinx Research Institute, San Diego, CA, USA; ^3^ Research and Toxicology Department, Humane Society International, London, UK; ^4^ Cellular Dynamics International, Madison, WI, USA; ^5^ CAAT, Johns Hopkins Bloomberg School of Public Health, Baltimore, MD, USA; ^6^ College of Sciences, University of Texas at San Antonio, San Antonio, TX, USA; ^7^ Department of Neurology, Mayo Clinic, Rochester, MN, USA; ^8^ Centre for Biomedical Sciences, School of Biological Sciences, Royal Holloway University of London, Egham, UK; ^9^ Division of Clinical Education, Midwestern University, Glendale, AZ, USA; ^10^ Division of Neurobiology, Barrow Neurological Institute, Phoenix, AZ, USA; ^11^ Department of Psychology, University of Quebec/Montreal, Montreal, Canada; ^12^ Section of Nutrition and Nutritional Epidemiology, Department of Internal Medicine, Rush University, Chicago, IL, USA

**Keywords:** Alzheimer disease, animal models, human methods, induced pluripotent stem cells, computational models, Gerotarget

## Abstract

Much of Alzheimer disease (AD) research has been traditionally based on the use of animals, which have been extensively applied in an effort to both improve our understanding of the pathophysiological mechanisms of the disease and to test novel therapeutic approaches. However, decades of such research have not effectively translated into substantial therapeutic success for human patients. Here we critically discuss these issues in order to determine how existing human-based methods can be applied to study AD pathology and develop novel therapeutics. These methods, which include patient-derived cells, computational analysis and models, together with large-scale epidemiological studies represent novel and exciting tools to enhance and forward AD research. In particular, these methods are helping advance AD research by contributing multifactorial and multidimensional perspectives, especially considering the crucial role played by lifestyle risk factors in the determination of AD risk. In addition to research techniques, we also consider related pitfalls and flaws in the current research funding system. Conversely, we identify encouraging new trends in research and government policy. In light of these new research directions, we provide recommendations regarding prioritization of research funding. The goal of this document is to stimulate scientific and public discussion on the need to explore new avenues in AD research, considering outcome and ethics as core principles to reliably judge traditional research efforts and eventually undertake new research strategies.

## INTRODUCTION

On April 17^th^ 2015 the Physicians Committee for Responsible Medicine (http://www.pcrm.org/) held a roundtable with expert researchers on Alzheimer disease (AD) and human-based research approaches from the United Stated and the United Kingdom, to discuss why and how the AD research community should adopt human-based research strategies to overcome the increasing prevalence of AD in the 21^st^ century. The major goals of the roundtable were: (1) to discuss the relevance of human-based models and tools for investigating AD pathophysiology at multiple levels of biological complexity, taking human relevance into account; (2) to formulate strategic recommendations as potential guidelines for determining research funding priorities in the field of AD research. In the present document we describe the major discussion outcomes of that meeting. We also reflect on how these recommendations fit in with current, quickly evolving, scientific and public policy efforts.

It is important to note that roundtable participants sometimes expressed different opinions regarding the discussed topics. While some felt that the first step should be to reduce animal models, others felt that current techniques already offer vast, powerful and unexplored pathways to study AD, and that sufficient alternatives already exist to fully proceed with human-based research. However, all participants agreed that there is now a range of new techniques and research directions that have been under-explored and need to be supported through changes in public funding and research priorities.

## THE AD RESEARCH PARADIGM IS FAILING: MAIN FACTS SUPPORTING THIS PREMISE

Alzheimer disease (AD) represents the most common cause of dementia, accounting for 50-75% of all dementia cases [1, 2]. The number of persons affected by AD in the United States is expected to almost triple by 2050, reaching 13.8 million [3]. Despite intense research efforts, the mechanisms of action of both protective and causative factors for AD are still not clearly understood. In the last ten years no new drugs have been released and existing drugs only stabilize symptoms temporarily in some patients, but do not slow progression of the disease [4, 5]. This defeat is reflected by the dramatically high clinical failure rate (99.6%), which is the highest among biomedical research fields [6–8]. One bright prospect is that the occurrence of dementia has recently been reported to be stabilizing in Western Europe, but this has been primarily attributed to preventative approaches and improvements in living conditions [9]. Analogously, the Framingham Heart Study has reported a decline of both vascular risk factors and the risk of dementia associated with heart failure, stroke, or atrial fibrillation over the course of thirty years [10].

Much of the traditional AD research has been based on the use of animal models, often transgenic (Tg) and inbred mice, in an effort to recapitulate genetic and pathological traits of human disease [11]. However, Tg animals, despite presenting several of the typical AD traits, such as amyloid β (Aβ) formation, neuritic plaques, neurofibrillary tangles (NFT), gliosis, synaptic alterations and signs of neurodegeneration, do not develop the clinicopathological complexities of human AD [12–15]. Moreover, treatments that seem to work in such models have not translated to humans [11, 16–18]. This indicates the existence of a clear disconnection between the (animal) model and the human condition [17] that is not taken into sufficient account by investigators. Another issue with animal models is that they might also be generating false negative data, leading to the exclusion of compounds from clinical studies that could be effective in humans.

**Figure 1:**
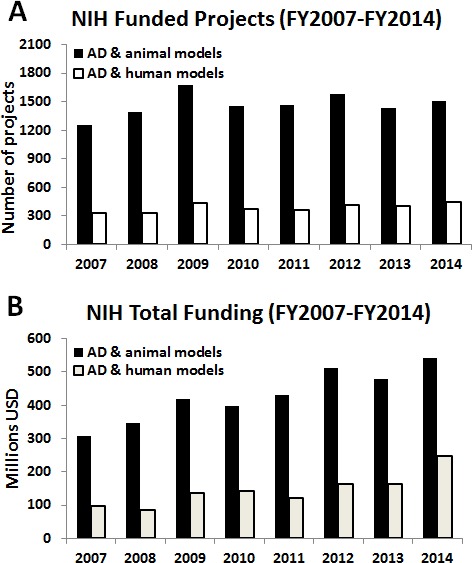
Bar graphs reporting the absolute numbers of AD-related projects focused on the use of animal models (black bars) *vs* projects accounting only for human-relevant models/methods (white bars) **A.** and relative funding **B.**, provided by the NIH from fiscal year (FY) 2007 to 2014. Analysis has been done usinghttp://projectreporter.nih.gov/reporter.cfm (as of July 6^th^ 2015), project search was limited to ‘project terms’. List of applied keywords per category: AD & animal models: Alzheimer AND (“primate” OR “primates” OR “monkey” OR “monkeys” OR “macaca” OR “macaque” OR “marmoset” OR “vervet” OR “cercopithecus” OR “cynomolgus” OR “tamarin” OR “dog” OR “dogs” OR “canine” OR “canines” OR “canis” OR “feline” OR “felines” OR “felis” OR “guinea” OR “rabbit” OR “rabbits” OR “mouse” OR “mice” OR “porcine” OR “pig” OR “pigs” OR “ovine” OR “sheep” OR “rattus” OR “rat” OR “rats” OR “mus” OR “mice” OR “mouse” OR “mammal” OR “fish” OR “zebrafish” OR “hamster” OR “rodent” OR “animal model” OR “animals” OR “animal” OR “xenopus” OR “caenorhabditis elegans” OR “c. elegans” OR “drosophila melanogaster” OR “drosophila” OR “lamprey”). AD & human models: Alzheimer AND “human” AND (“stem cells” OR “induced pluripotent stem cells” OR “iPS” OR “imaging” OR “PET” OR “MRI” OR “computational” OR “prevention” OR “preventive strategy” OR “clinical study” OR “clinical” OR “clinical trial” OR “patient”) NOT (“primate” OR “primates” OR “monkey” OR “monkeys” OR “macaca” OR “macaque” OR “marmoset” OR “vervet” OR “cercopithecus” OR “cynomolgus” OR “tamarin” OR “dog” OR “dogs” OR “canine” OR “canines” OR “canis” OR “feline” OR “felines” OR “felis” OR “guinea” OR “rabbit” OR “rabbits” OR “mouse” OR “mice” OR “porcine” OR “pig” OR “pigs” OR “ovine” OR “sheep” OR “rattus” OR “rat” OR “rats” OR “mus” OR “mice” OR “mouse” OR “mammal” OR “fish” OR “zebrafish” OR “hamster” OR “rodent” OR “animal model” OR “animals” OR “animal” OR “xenopus” OR “caenorhabditis elegans” OR “c. elegans” OR “drosophila melanogaster” OR “drosophila” OR “lamprey”)

An examination of current methodological approaches, suggests a bias in the peer-review process in favor of using these animal models *versus* alternative approaches. Specifically, the number of projects - and funds - based on animal models supported by the U.S. National Institutes of Health (NIH) over the last eight years is much higher than the number of research projects focused on the use of human-based models and methods (e.g., human-derived (stem) cells, neuroimaging, computational models, prevention, clinical studies, etc.) (Figure 1). Although efforts such as the National Alzheimer's Project Act — which has dramatically boosted resources for AD and related dementias (ADRD) research — are beginning to prioritize human relevant approaches and attempting to address the multifactorial and multi-etiology of dementia there remains a strong bias towards animal research approaches. This bias can be seen throughout reports and recommendations in the strong linking of “animal research” with “basic research”. For example, in the 2016 update of draft prioritized recommendations of the ADRD, the implementation of the recommendation from Session 6, Focus Area 1: Basic Mechanisms and Experimental Models - “Develop next generation experimental models and translational methods for VCID (vascular contributions to cognitive impairment and dementia)” six out of nine recommendations explicitly call for the development of of animal models [19]. In conflating the concepts of animal research and basic research there is a failure to recognize the important development that basic laboratory research is increasingly being performed entirely without the use of animals. Indeed, without the critical acknowledgement of the failure of past animal paradigms and the promise of new approaches, new attempts at making progress in basic AD research will be severely hampered.

Beyond laboratory models, several lifestyle-related risk factors have been shown to play key roles in the onset and progression of AD, yet research support in theses domains remains disproportionately low, with only a 3.4% of average annual funding supported by the National Institute on Aging (NIA) for prevention in 2010-2012 [20]. Although advancing age is clearly considered the main risk factor for developing AD [21–23], nutritional factors [24], low levels of physical activity [25, 26], reduced cognitive stimulation [27], socioeconomic status and educational attainment [28–30] are all directly related to AD risk. Furthermore, poor sleep quality [31–33] which is known to positively correlate to early Aβ deposition [34, 35], air pollution [36], smoking [37], intake of metals [38–40], pesticides and insecticides [41, 42] as well as metabolic-related dysfunctions [43, 44] have all been described as possible risk factors.

These data indicate that - rather than an independent health condition - AD should be reinterpreted as a complex multifactorial syndrome. Understanding this complexity is clearly critical for designing intervention strategies aimed at preventing or ameliorating early symptoms of AD. Despite this knowledge and massive potential social impact, longitudinal clinical studies focused on prevention are very poorly supported in the U.S. (only ~ 7%-9% of the $30 billion NIH total discretionary budget) [20]. For all these reasons there is an urgent need to rethink current research funding strategies to directly target human relevance and disease causation.

## ADDRESSING HUMAN RELEVANCE IN AD RESEARCH WITH THE USE OF ALTERNATIVES TO ANIMAL EXPERIMENTS

Recent developments have brought about a staggering array of research approaches that are offering bold new ways to study human brain aging and are yielding profuse and meaningful human relevant data. These techniques include: (1) several human-based models focused on the use of patient-derived cells, such as induced pluripotent stem cells (iPSCs) and neuronal and glial cultures, (2) multiple ‘omic’ technologies (e.g., genomics, proteomics, lipidomics, transcriptomics, metabolomics, etc.) resulting from overall analyses of biological samples by high-throughput analytical approaches and databases, (3) computational analytical approaches and (4) novel neuroimaging readouts [17, 18, 45].

Given the need to integrate the huge amount of incoming data, comprehensive multi-scale and systems biology approaches are becoming fundamentally important. These approaches must take into account all the different levels of biological complexity (including population, individual, organ/tissue, cellular, protein, and gene level), thereby allowing for the elucidation of disease-related adverse outcome pathways (AOPs), as already envisioned in toxicology [46] and proposed for AD research [17]. Within this new framework it is becoming increasingly possible to not only determine the effects of an exposure to a given compound (for instance, pollutants possibly implicated in the onset of AD) but also to investigate how these effects are induced [47, 48]. Defining which signaling pathways are perturbed at early stages of AD (i.e., the AD-related pathways) might help predict long-term effects and sequelae. For this reason, multiscale AOPs should become the core of the new paradigm in AD research. Investigating AD-related multiscale AOPs could allow researchers to link environmental and genetic causes with outcomes at individual/body level [17].

A number of cellular *in vitro* models of AD and human-based methods can already take into account different levels of biological complexity (Table 1). For example, iPSCs have been widely applied in AD research [49–53] and can be used to: (i) assess clinical candidate drugs on human brain cell types; (ii) conduct phenotypic screening of compounds that modulate or normalize disease phenotype; (iii) conduct target-based screening if candidate genes are identified; (iv) compare genetically-diverse panels; or (v) select or stratify participants of clinical trials based on their genetic backgrounds and/or phenotypic traits.

Moreover, human-based intervention trials focused on nutrition, physical activity, and cognitive training are particularly relevant to preventing AD and cognitive decline. These trials have proven to be the most effective strategies to reduce AD symptoms [20, 25, 26, 54–59].

In order to stimulate the creation of multifactorial approaches to AD, global efforts have been made to improve access and discussion online for researchers. In recent years, common platforms, such as CLIR (Collaborative Laboratory Integrated Reports), developed at the Mayo Clinic (https://clir.mayo.edu/), have been shown suitable to create groups of interest and propose multidisciplinary team approaches, allowing comparisons among different sub-populations, different ages and different treatments. At the clinical level, databases, such as the Laboratory of Neuroimaging - Image Data Archive (LONI-IDA), provide user access to de-identified data from positron emission tomography (PET), magnetic resonance imaging (MRI), cognitive data sets and biomarkers. These interfaces represent a large step forward in maximizing the impact of these data.

**Table 1:** Human-based studies, models and readouts suitable for AD research

Human-based models/tools	Characteristics and applicability	Biological complexity level
Epidemiological studies, randomized clinical trials	To assess the complex interrelations of risk factors and ameliorating influences including: environmental triggers, genetic susceptibility, sex, gender, diet, physical activity, co-occuring conditions (e.g., diabetes), cognitive engagement, social interactions and other cultural factors.	Population, individual
Human ex vivo tissue	Healthy and diseased brain tissues with short post-mortem intervals, standardized preparation, accessible samples and data. To account for patient heterogeneity and study cellular and structural pathologies. To aid in the validation of biomarkers and to refine analysis of factors involved in disease progression	Individual, whole brain
Neuroimaging techniques (e.g., MRI, PET, MRI tractography)	To study human brain anatomy through 2D and 3D images in *in vivo* and *ex vivo* studies. To refine AD diagnosis and uncover early markers of disease, understand longitudinal structural development of AD, assessment of treatment effects, construction of brain atlas/connectome	Individual, whole brain
Connectomics, PBPK, and PD studies, IVIVE,	To define kinetics and dynamics of environmental factors (e.g., compounds, nutrients) exposure and to predict their long term effects in relation to AD. To assess the efficacy of compounds for AD treatment	Individual, whole brain
Microfluidics/organ-on-chip	To investigate tissue complexity, assess effects of possible therapeutic compounds.	Tissue, whole brain
Patient-derived samples: CSF, blood/plasma, fibroblasts, lymphocytes	To define early biomarkers of AD, to generate xeno-free iPSCs.	Tissue
3D models, organoid systems (e.g., iPSCs, NSCs)	To mimic physiology of the brain tissues. Suitable depending on the research goals.	Cell, Networks, Organoids
Early, familial and late-onset AD patient-iPSCs and their differentiated functional derivatives (2D and 3D)	Glutamatergic & cholinergic neurons and astrocytes. iPSC-neurons show AD phenotypic traits consistent with the Aβ tau hypotheses after limited time in culture (e.g., elevated Aβ production, increased levels of p-tau) and responsiveness to β and γ secretase inhibitors. Genome-editing technologies (e.g., ZFN, TALENs, and CRISPR/Cas9) can be used to add, disrupt or modify the sequence of specific genes related to AD, measure their impact on human iPSC-derived neurons and, ideally, design patient tailored treatments. To identify disease pathways & drug targets, & assess therapeutic compounds.	Cell, Assemblies, Networks
Synchrotron x-ray fluorescence imaging	To define bio-metals distribution and concentrations in the human brain affected in AD. To characterize the metallo-relationship of plaques and tangles, volumetric reductions in brain regions in AD.	Multi-scale: Sub-cellular to Individual, whole brain
Omics: transcriptomics, proteomics, lipidomics, metabolomics, exposomics, nutrigenomics, nutrigenetics, genomics, epigenomics	To assess signaling pathways, epigenetic, genetic mutations, gene expression & lifetime exposures	Protein, gene, individual
Computational modeling	Can be applied at any of the above levels to investigate the causal relations, illuminate underlying mechanisms and to help predict outcomes of interventions in relation to AD at single and multiple scales	Ranges from gene to neural population dynamics

**Abbreviations:** CSF, cerebrospinal fluid; iPSCs, induced pluripotent stem cells; NSCs, neural stem cells; MRI, magnetic resonance imaging; PET, positron emission tomography; PBPK, physiologically based pharmacokinetics; IVIVE, *in vitro-in vivo* extrapolation; PD, pharmacodynamics; ZFN, zinc-finger-nucleases; TALENs, transcription activator-like effector nucleases; CRISPR/Cas9, clustered regularly-interspaced short palindromic repeats/CRISPR-associated protein-9 nucleases.

As alternatives to the use of traditional mammalian species, some non-mammalian/non-vertebrate models, such as *Dictyostelium discoideum*, have also be applied to undertake new directions in basic research [60, 61] and define the role of previously unexplored proteins/molecules. Taking into account their biological limitations (e.g., they cannot effectively be used to mimic the large-scale anatomical and behavioral aspects of an aging human brain), these non-mammalian models are relatively easy to handle, cost effective, and can be manipulated to express AD-related human genes/proteins [61]. providing innovative approaches to research in this area.

The availability of a range of approaches and collaborative tools is an important development in AD research. Obviously, the use of a given model should be driven by the specific research objectives, whether they be basic research or translational. In light of the fact that a unique model suitable to tackle all aspects of AD does not (and may never) exist, different models might be suitable to cover the many different aspects of the disease depending on the level and mechanism being investigated. For this reason, in an effort to gather a global picture of the environmental/lifestyle risk factors, the etiopathological mechanisms of the disease and define possible preventive, intervention and pharmaceutical strategies, the creation of a multidisciplinary team approach in AD research, combining different expertise, should be mandatory. In line with this, some research initiatives have been undertaken in an effort to define correlations among Aβ formation, neuroanatomy, cognitive and lifestyle factors [62, 63].

## LIMITATIONS OF ALTERNATIVE APPROACHES AND STRATEGIES TO OVERCOME THESE LIMITATIONS

Despite the great potential of new human-based approaches and non-mammalian models, their broad applicability and reliability is currently hampered by some limitations. It is essential to clearly recognize these constraints and define strategies to overcome them (Table 2).

**Table 2:** Limitations of alternative models and methods and strategies to overcome these limitations

Human-based models/tools	Limitations	Strategies to address limitations
Epidemiological studies, randomized clinical trials	Inability to determine causality due to potential multiple interacting and confounding factors Difficult to compare studies designed according to different inclusion/exclusion criteria	Comprehensive assessment of multiple behaviors and risk factors and complex multivariate analyses to address conjoint confounding and effect modification. Application of machine learning and other techniques capable of non-linear and high-dimensional pattern recognition in large data sets.
		Possibility to create multi-center collaborations, taking advantage of common platforms
		Multiple intervention studies to test treatment effects in different types of populations
Patient-derived samples: CSF, blood/plasma, fibroblasts, and postmortem AD and control brain tissues	Storage and analytic methods are often not standardized, preventing inter-lab comparisons Poorly preserved brain tissues and long postmortem delays Samples are often not readily available for test for reproducibility and validation	Creation of multi-center collaborations to standardize methods & optimize distribution: e.g., 2-3 nationwide brain banks centers of excellence, with 24/7 autopsy services, short postmortem delays (2-3 hours maximum) and with standardized neuropathological protocols. Digitize neuropathology finds using standardized methods and creating an open-access database for additional analysis.
Neuroimaging techniques (e.g., MRI, PET, MRI tractography)	High costs; sometimes weak correlations between measures and clinical manifestations; sometimes difficult to quantify	Consider large-scale studies to improve correlations between imaging measurements and clinical manifestations
Synchrotron x ray fluorescence imaging	Requires *ex vivo* or post mortem brain tissue	Integrate this technology with other neuroimaging tools
Microfluidics/organ-on-chip	Some limitations with regard to transport and diffusion of nutrients and oxygen; individual organs, kept in isolation	Increase investment in research and development. Complement these technologies with neuroimaging data and/or other omics data sets
3D models (e.g., iPSCs, NPCs)	Not applicable for all purposes	Integrate 3D models with 2D models depending on applications and research goals
AD patient-iPSCs and their differentiated functional derivatives	Generating high-quality iPSCs is expensive and time consuming; a limited number of AD iPSC lines have been generated and thoroughly characterized so far	Cost is dropping over time; several entities (e.g., CIRM, NYSCF, etc.) are funding the development of hundreds of iPSC lines from AD patients
	They might be not fully representative of the complex physiology of the brain and/or of AD pathophysiology	Possibility to create co-culture systems with human microglial cells. Genome-editing technologies can be applied to create mutations related to the AD genetics, measure their impact on patient iPSC-derived neurons and design patient tailored treatments.
	Different reprogramming and QCs have been used, so comparisons between labs are difficult to make at this time	Several entities (e.g., CIRM, NYSCF, etc.) could standardize reprogramming methods allowing inter-lab comparisons
	Challenges with regard to penetrance, cell purity, degree and type of differentiated cells generated from iPSCs	Need to harmonize QC standards, which would be more feasible with the participations of dedicated entities
	Traditional reprogramming methods (e.g., integrating lentiviruses) and xeno-contamination might have affected the phenotype of the lines	Develop and adopt xeno-free techniques with non-integrating reprogramming vectors
	Epigenetic signatures of the somatic cell of origin might be retained in the reprogrammed iPSCs (NB: evidence that epigenetic traits get lost upon long term culture)	Possibility to directly reprogram fibroblasts into neurons
		Possibility to reprogram post-mitotic neurons and frozen brain tissue samples into iPSCs (to retain the neuronal epigenetic and pathologic background)
	iPSCs metabolic profile has not been investigated enough (which has special relevance in AD research)	Define QC metrics to establish metabolic features of iPSCs
	Still not clear how long iPSC-derived neurons should be kept in culture in order to mimic late-onset AD neurons and tissue pathophysiology; possible issues with the loss of aging-related transcriptional signatures and features.	Use AD brain tissues as benchmark models to define QC metrics suitable to assess neuronal and pathological features of differentiated iPSCs. Overexpression of aging-related genes (e.g., progerin) might help model AD in a dish. Direct conversion of aging donors' fibroblasts into neurons (iNs) can help retain aging-related transcriptional signatures.
	2D and 3D iPSC cultures might be characterized by different biological/cellular/molecular features and generate different responses	Define QC metrics to establish features of 2D vs. 3D iPSC cultures
	Not clear if AD-derived fibroblasts might be proven as suitable as their reprogrammed counterparts (i.e. iPSCs) to define molecular/cellular features of AD (e.g., metabolic profiles)	Define QC metrics to establish features of AD-derived fibroblasts vs AD-derived reprogrammed iPSCs
Non-mammalian/invertebrate models of AD	More phylogenetically distant from humans than mammalian species; might lead to intermediate validation steps in mammalian (non-human) species	Consider their suitability for basic research effort; less time consuming and less expensive than traditional animal models
		Investigate directly in human *ex vivo* tissues/cultures (rather than animals) to assess preclinical data (applying microdosing analysis)
PBPK and PD studies, IVIVE	PBPK, PD and IVIVE are currently applied mainly in toxicology.	Possibility to establish dedicated consortia with a multi-disciplinary approach (e.g., combining medical research and toxicology expertise).
Connectomics, computational analysis and modeling	Connectomics still in early development. Resolution too low. Very large data sets. Computational models are often restricted to simply mimicking observed phenomena and have no predictive value.	Develop techniques to study both individual and large cohorts necessary to recognize significant patterns. Increases in resolution, computational power and large-scale analysis algorithms are all rapidly improving. Encourage move to foundational computational simulations that explore the basic effects of cellular, network and system factors in aging and dementia. Use to elucidate and predict previously unrecognized changes in anatomy, physiology and cognition.
Various other omics: transcriptomics, proteomics, lipidomics metabolomics, exposomics, nutrigenomics, nutrigenetics, genomics, epigenomics	High costs	Costs of analysis are reducing. Possibility to establish dedicated consortia with a multi-disciplinary approach (e.g., combining molecular biology and biostatics expertise)

Abbreviations: CSF, cerebrospinal fluid; iPSCs, induced pluripotent stem cells; QC, quality control; NSCs, neural stem cells; MRI, magnetic resonance imaging; PET, positron emission tomography; PBPK, physiologically based pharmacokinetic; IVIVE, *in vitro-in vivo* extrapolation; PD, pharmacodynamics

### AD-derived iPSC models

With specific regard to iPSC-derived models of AD, it is generally recognized that generating high-quality iPSC lines is still expensive and time consuming. In addition, only a limited number of AD iPSC-derived lines have been generated and thoroughly characterized so far. These AD-related iPSC studies have used different programming and quality control methods, as well a variety of somatic cell types. These differences in protocol make inter-laboratory comparisons difficult at this time. Moreover, the reprogramming mechanism used to generate older iPSC lines, are often based on the use of integrating lentiviruses and retroviruses, which may have caused insertional mutagenesis [64, 65]. Many approaches remain xeno-contaminated. For this reason, to minimize these issues, current and future reprogramming methods should aim to be xeno-free and based on the use of non-integrating reprogramming vectors or entirely vector-free approaches.

Nevertheless, the iPSC approach holds enormous potentials and the rapidly expanding research field is already tackling these limitations. For example, the production cost is progressively dropping, and several entities, such as the California Institute for Regenerative Medicine (CIRM,https://www.cirm.ca.gov/) and the New York Stem Cell Foundation (NYSCF,http://nyscf.org/) are currently funding the development of hundreds of iPSC lines from both early- and late-onset AD patients that will be available globally at low cost to investigators. It will now be important to generate dedicated and accessible bio-banks for the collection and distribution of AD patient-derived fibroblasts or peripheral blood cells for reprogramming purposes, accounting for both late- and early-onset AD, mild cognitive impairment, and healthy elderly donors. These samples should be made available to the scientific community whenever required to facilitate inter-laboratory reproducibility, data validation and outline correlations between patients' clinical history and patient-related cellular and molecular data sets, which might help develop novel therapies. In particular, the collection of late-onset, sporadic AD patient-derived fibroblasts for the generation of late-onset AD iPSCs will be critical in providing insight into late-onset AD pathology, which represents the majority of AD cases (~95%), as compared to early-onset AD (representing ~5% of all AD cases) [23, 66]. These collection efforts should pay particular attention to the language of the informed consent forms used in donor recruitment in order to ensure patient protection as well as the broadest possible use of the samples by both in academic and commercial research and clinical usersapplications.

Several aspects of the generation of iPSCs as well as their use in studying AD include the “epigenetic memory”, the types of reprogrammed cells used for studying the etiology of AD, and the ability of iPSCs to mimic AD pathophysiology. With regards to this “epigenetic memory” phenomenon, there is evidence that the epigenetic signatures of the somatic cells of origin might be retained in the reprogrammed iPSCs. As a consequence, iPSCs might preferentially generate derivatives of the donor somatic cell type [67, 68], inadvertently skewing results. Possible strategies to overcome this limitation might be to directly reprogram fibroblasts into nervous system cell types [69, 70] or, in order to retain the epigenetic background of neuronal cells, to reprogram post-mitotic neurons into iPSCs [71]. iPSCs have been successfully obtained by reprogramming frozen non-cryoprotected dural tissue samples (stored at −80°C for up to 11 years), which allowed for generating iPSCs with confirmed pathology even from AD patients with rare genetic variants [72]. Nevertheless, there is evidence that iPSCs lose epigenetic traits during long term culture [73], which might be considered either as a positive aspect (as the epigenetic memory of somatic cells of origin might be mitigated) or a negative aspect (in light of the fact that AD patient epigenetic signatures might also be lost over time).

Additionally, it is also unclear whether AD-derived fibroblasts might be proven as suitable as their reprogrammed iPSC counterparts to define some of the molecular/cellular features of AD. For instance, using AD patient-derived fibroblasts or other cell types, such as peripheral blood mononuclear cells, might be sufficient to detail some AD-related genetic, epigenetic and/or metabolic features, avoiding all the reprogramming steps and the overall time consuming neuronal differentiation process. For all these reasons, establishing appropriate quality control metrics, accounting for gene expression analyses and quantifications of protein/biomarker levels will help define the expandability of an iPSC model for a given purpose and harmonize data interpretations, allowing inter-laboratory comparisons, as already envisioned and practiced in toxicology studies [74].

Furthermore, there are some challenges with regard to penetrance, cell purity, degree and type of differentiated cells that can be generated from iPSCs that need to be taken into account when developing new models. In terms of the future directions of iPSCs in AD, greater consideration to the metabolic profile would be advantageous. The majority of studies published so far have not extensively investigated the iPSC metabolic profile; however, several lines of evidence indicate that AD should be studied as a complex systemic/metabolic dysfunction, correlated to metabolic syndrome [43], hypometabolism, oxidative stress, and modifications of the glucose-fatty acid cycle [75].

Beyond issues of epigenetic memory and metabolism, it remains unclear how long iPSC-derived neurons should be kept in culture in order to mimic the development of late-onset AD neuronal cells and tissue pathophysiology [76]. In general, modeling aging and neurodegenerative disease, like AD, in differentiated human neurons, such as those derived from AD patient iPSCs, can be a challenging task. Often neuronal cells cultured *in vitro* do not retain the aging-associated transcriptional profile and phenotype, which represents a major issue when modeling late-onset disorders, such as late-onset AD. There are some possible ways to “age” human neurons in a dish. In particular, the overexpression of the premature aging-related gene s, such as progerin, has been shown suitable to model Parkinson's disease in iPSCs and might be possibly applicable also for AD-iPSCs [77]. Alternatively, the direct conversion of aging donors' fibroblasts into neurons (namedcalled induced neurons, or iNs), avoiding cells reprogramming toward the an embryonic phenotype, has been shown promiseing; iNs were found to retain an aging-related transcriptional signature (i.e., decline of the nuclear transport receptor RanBP17) when compared to iPSCs and their neuronal derivatives [78].

Additionally, genome-editing technologies, such as the zinc-finger-nucleases (ZFN), the transcription activator-like effector nucleases (TALENs), and the clustered regularly-interspaced short palindromic repeats/CRISPR-associated protein-9 nucleases (CRISPR/Cas9) can now be used to overcome variability in human genomes. Although still in development, these genome-editing technologies can already be used to add, disrupt or modify the sequence of specific genes related to AD and measure their impact on human iPSC-derived neurons [79]. In particular, these nucleases can induce guided DNA breaks, which can be repaired by homologous recombination with a donor vector carrying a desired point mutation or gene, in order to better model the disease *in vitro* and, ideally, design patient tailored treatments [80–82].

Moreover, even as researchers are using cell lines to address the complexities of developing a robust two-dimensional *in vitro* model for AD research, others are taking it a step further by working towards three-dimensional iPSC cultures. This is important because two-dimensional cultures of iPSCs seem to be characterized by considerably different biological, cellular, and molecular features as compared with their three-dimensional counterparts [51]. The implications of the differences in responses upon exposure to potential therapeutic compounds in two-dimensional *versus* three-dimensional models still need to be elucidated and their biological relevance assessed. Despite these limitations and open questions the added dimension provides an entirely new platform to investigate pathology and therapeutics (Table 2).

### Microfluidics/organ-on-chip systems

Beyond iPSCs models, microfluidics/organ-on-chip systems have been created in an effort to simulate *in vitro* human organ and tissue biology and function. These models combine different cell types in specific 3D culture systems [83, 84] and might be useful to test novel therapeutic compounds in human physiological-like systems. However, despite their potential applicability, these technologies are still in their infancy and require further validation. Additionally, these models, regardless of their level of optimization, remain fundamentally disembodied and thus cannot capture the full complexity and physiological function of a living organism. For this reason, direct clinical studies, including neuroimaging data and various omics data sets, that incorporate the full richness of human cognitive, environmental and social interactions will be required to complement and interpret information derived from these *in vitro* models (Table 2).

### Non-mammalian models of AD

Non-mammalian/invertebrate models of AD (e.g., *Dictyostelium discoideum*, *Drosophila melanogaster*, etc.) were previously judged poorly relevant from a biological standpoint and for this reason less worthy of funding compared to mammalian species phylogenetically closer to humans (Table 2). Nevertheless, it's worthwhile considering the limited “return on investment” that has been gained after extensively funding translational research projects focused on the use of traditional mammalian models, in particular mice. The over-reliance on the use of animals, together with the lack of implementation and optimization of human-based models, have contributed to the current clinical attrition rate in AD translational research [6, 8, 16].

Although non-mammalian species can be used to define basic disease mechanisms, human-based cell models, such as AD-iPSC neuronal cell cultures and human-based organ-on-chip systems, could also be applied for pre-clinical drug discovery.

One approach that has been suggested is to validate data obtained in non-mammalian models in a small number of animals before moving to human tissues/cultures or clinical trials, thereby contributing to a reduction of the use of animals, according to the 3Rs principle envisioned in toxicology and biomedical research [85]. However, it should also be considered that intermediate validation steps in non-human mammals might generate false negative results, possibly invalidating results that might actually be proven valuable in human settings. Moreover, the assumption that non-mammalian species require intermediate validation steps in mammalian non-human models before translating obtained data into humans largely remains unquestioned. However, data obtained in non-mammalian and non-animal models can be validated directly and more effectively in human *ex vivo* tissues/cultures and/or postmortem tissue. This is already being done for some rare diseases, using microdosing analyses [86]. Most importantly, the rapidly expanding availability of direct human assays, imaging and clinical data collection techniques is increasingly rendering animal models of all scales unnecessary.

### Post-mortem AD brain tissues

Post-mortem AD brain tissues are important biological resources for AD research, from which the major AD therapies were discovered [87]. It is also critical as a validation resource with which to assess discoveries from cell culture and animal models. They represent an invaluable resource to conduct neuropathology studies, spanning from morphology, connectivity, cellular, molecular and genome perspectives. Moreover, as clinical studies of AD attempt to discover earlier and more sensitive biomarkers, neuropathology studies remain the key reference for validation [88].

However, brain tissue samples are often of suboptimal quality, due to long postmortem delays and inappropriate postmortem handling and storage. High quality brain tissue from normal control subjects is particularly scarce. Some aspects of protein function, phosphorylation [89], RNA integrity and the aforementioned epigenetic modifications are strongly altered by postmortem delay, freeze-thaw cycles and even by freezing itself [90]. For these reasons, much of the currently available brain tissue, while being useful to conduct morphological studies and assess robust disease biomarkers, may be unsuitable for many molecular studies [91, 92]. Nevertheless, microRNA analysis of AD-affected temporal lobe neocortical tissues collected in short post-mortem interval of about 1 hour can provide important starting points for examining specific AD alterations [93].

It has to be considered that limited availability of post-mortem tissue and differing collection and preservation protocols make projects requiring very large subject numbers, as well as inter-laboratory replication studies difficult or impossible to perform. For these reasons, the creation of multi-center collaborations and bio-banks would greatly increase the ability of researchers to make the most of these resources (Table 2). However, it would be counter-productive to simply replicate existing brain bank networks, such as those of the NIA Alzheimer's Disease Centers (https://www.nia.nih.gov/alzheimers/alzheimers-disease-research-centers) or BrainNet Europe (http://www.brainnet-europe.org/) as these have not been able to rapidly and systematically provide autopsies or sufficient numbers of normal control brains. A more targeted approach would be to provide proportionately greater funding to a small number of specialized centers, allowing them to meet these critical needs. These centers should allow a streamlined system of sharing, improve timing of distribution, increase the quality of the available materials, harmonize tissue collection standards and provide better correlations between the pathology and neuroimaging patient data. A more ambitious, yet potential ground-breaking step for neuropathological approaches would be to digitize research data and make them openly available along with detailed tissue collection and analysis protocols. A model for this could be the Alzheimer's Disease Neuroimaging Initiative (ADNI), which provides in-depth information in their neuroimaging, biomarkers and genetics data collected from large multicenter collaborations [94]. This level of sharing and standardization would maximize the usage of these precious tissue resources and accelerate the improvement of neuropathologic approaches by increasing interactions between investigators.

### Neuroimaging

Novel *in vivo* imaging readouts, such as PET and ultra-high-field MRI are currently available to diagnose AD [95, 96] and have been successfully applied to assess *in vivo* the effects of specific nutritional interventions [54–56]. Importantly, neuroimaging readouts have been critical to discover commonalities of neuroanatomical features shared by AD, type-2 diabetes and the metabolic syndrome [45, 97–99], pathologies that have been shown to be highly interconnected [100–102]. Moreover, human connectomics enabled by techniques such as MRI tractography, allows for the reconstruction of 3D neuronal networks, brain anatomy, and AD-related neuroanatomical modifications [103–105].

As is the case with many new approaches, future neuroimaging technologies will need to overcome the sometimes prohibitively expensive development and operational costs. In addition, researchers will need to improve the clarity of the identification of correlations between retrieved measures and AD-related clinical manifestations, as well as devise methods to deal with the complex data sets to improve quantification of imaging features. Despite current limitations, even existing technologies represent essential tools to support human-relevant AD research approaches, and significant extension of large-scale clinical studies using current techniques should be encouraged to help improve correlations between imaging measurements and clinical manifestations (Table 2).

### ‘Omics’ technologies and computational models

Despite their relatively high costs, high-throughput technologies, such as proteomics, lipidomics, metabolomics, epigenomics, and genomics, are currently applied to define the molecular mechanisms underlying AD pathogenesis [106–111].

Additionally, computational models, such as *in vitro-in vivo* extrapolation (IVIVE), physiologically based pharmacokinetic (PBPK), and pharmacodynamics (PD) modeling, are currently applied in the field of toxicology and regulatory testing, but might be suitable to define kinetics and dynamics of compound exposure, predict their long term effects in relation to AD [47, 48, 112], and assess therapeutic potential of novel compounds for AD treatment [113–115].

While these technologies are still under development and require further optimization, establishing dedicated consortia with a multi-disciplinary approach aimed at combining medical research with toxicology expertise might prove a winning strategy to speed the drug discovery process (Table 2). More broadly, computational approaches allow for unprecedented mining of data across levels. Similarly, beyond just data mining, computational simulations can explore the correlations and underlying mechanisms at levels ranging from the molecular to cellular to network, and even social scale. These simulation techniques offer great promise but remain largely underused.

## NEED TO PRIORITIZE HUMAN RELEVANT RESEARCH AND EXPLORE ALTERNATIVE RESEARCH AVENUES

Taking into account “human relevance” when addressing AD research efforts, funding agencies need to implement strategies that will encourage the use of these human-based models for AD research. The implementation of human-based methods will also contribute to minimize the use of sentient beings in biomedical research, as advocated by the NIH [116] and the public [117].

To this aim, requests for applications (RFAs) focused on the use of explicitly xeno-free human-based models and novel high-throughput technologies should be created. In particular, considering the potential of AD patient-derived iPSCs, study sections should include experts in the iPSC and reprogramming field, competent in evaluating research proposals focused on the use of iPSC models for AD.

Additionally, expansion of existing and creation of new centralized and open bio-banks providing iPSC lines and/or high quality post-mortem tissues should be encouraged and incentivized, allowing large scale distribution of biological samples to research institutes when needed. While entities distributing both healthy controls- and AD patients-derived iPSCs are already in place in Europe, the U.S. and Japan, iPSC lines are rarely fully characterized by providing entire genomic, epigenomic and patient phenotype data sets [118]. For this reason, efforts to assimilate best practice should be taken into account [118]. Interestingly, CIRM has recognized the value such genetic information adds to an iPSC line and released in early 2016 a RFA (DISC3.1) to characterize all 3000 lines in its repository. Additionally, pre-competitive and collaborative centralized distribution of iPSCs would help to increase biological sample quality and would allow for the creation of harmonized quality control standards for the use, characterization, handling and storage of biological material. Developing such guidance will accelerate inter-laboratory data comparison and validation. Again, consent forms need to allow such sharing.

Moreover, considering the important role played by lifestyle and environmental factors and the relevance of prevention to reduce the burden of AD [9], as also commented in the Leon Thal Symposium proceedings [119, 120], specific RFAs should be created to encourage investigation of early phenotypic traits of neurodegeneration and the implication of multiple networks (or “human disease pathways”) in neuronal failure at early stages of the disease. Amongst these networks, research on mitochondrial dysfunction, known to be an early event in AD progression [121–123], should be emphasized.

Learning from failure and ethics should be encouraged as a general attitude in research. Current and previous research efforts to study AD pathology in animal models and identify effective drug targets have not led to significant and effective prevention or disease modification in humans, so new avenues should be explored. In this regard, even re-evaluating the validity of traditional AD “gold standards” (i.e. the main diagnostic biomarkers of AD), such as presence of Aβ plaques and NFT, might help to hypothesize new therapeutic strategies. In particular, both Aβ and NFT are known to often appear early in time and, according to novel hypotheses, might actually not be considered as causative of AD but rather a result of AD pathology, which is often characterized by neuroinflammation as well as hypometabolism [43, 75, 124]. In this regard, negative results in science are not given enough consideration, frequently leading to their suppression during publication. However, negative results are crucial to establishing limitations of current research models [125], and defining the need for new research avenues.

The creation of pre-competitive consortia to judge the suitability of new models would be highly relevant. In this regard, pharmaceutical companies have shown a willingness to abandon obsolete models, investing resources in new human-derived paradigms involving pre-competitive consortia, such as the Cardiac Safety Research Consortium (http://cardiac-safety.org), the FDA's Critical Path Initiative (http://www.fda.gov/ScienceResearch/SpecialTopics/CriticalPathInitiative/ucm076689.htm), TransCelerate BioPharma (http://www.transceleratebiopharmainc.com), the international Serious Adverse Event Consortium (http://www.saeconsortium.org/) and larger international consortia, such as the Structural Genomics Consortium (http://www.thesgc.org). Analogously, in Europe, the EU commission has allocated 1BN euro into pre-competitive consortia (e.g., the Innovative Medicines Initiative is EU's largest public-private initiative,http://www.imi.europa.eu/). Thus, the paradigm shift to create more human-relevant standards for validation of translational research should be stimulated at all stakeholder levels.

Moreover, considering the relevance of prevention, it would be necessary to increase current research budgets allotted to preventive medical research and also to increase expertise in the field of education and nutrition in correlation to neurology.

## RECOMMENDATIONS TO GUIDE NEW FUNDING STRATEGIES

While it is important to provide appropriate funding to support research and speed the discovery process [126], currently available resources should also be better allocated, shifting the focus to prevention strategies with human relevance and to the use of human-based research methods, such as patient-derived iPSCs, computational methods, advanced brain imaging methods, epidemiological studies and human focused non-animal models. Given the important knowledge we already have regarding lifestyle-related factors (e.g., diet, exercise, environmental exposure, etc.), we recommend that public education and policy should be ramped up considerably with special attention to those who may contest these recommendation (e.g., fast food industry).

Considering the multi-dimensional nature of AD pathology, we believe that the time is ripe for a reevaluation of the current definitions of aging, cognition, and their relationship to a variety of biological, social, and environmental variables. Instead of examining a single variable or biomarker at a time, as has often been done in the past, it would be worthwhile to consider the interconnected implications of several genetic, epigenetic, morphological, environmental, behavioral and social factors in the onset and consolidation of AD.

This envisioned paradigm and the application of human-based models, in conjunction with large scale randomized clinical trials and multi-dimensional -omics readouts, will help revolutionize our knowledge of AD pathology and etiology, contributing to the creation of a more holistic perspective regarding AD in the context of aging and lifestyle.

The NIH ADRD Research Summit, held at NIH in February 2015, advocated for “a change in how the academic, biopharmaceutical and government sectors participating in Alzheimer's research and therapy generate, share and use knowledge to propel the development of critically needed therapies” [127]. In particular, the limitations of rodent models were highlighted [127]. In line with this, we propose a list of practical recommendations possibly suitable to guide current funding priorities in AD research, addressing human relevance. These recommendations, outlined in Table 3, are meant to be applicable to the NIH as well as any AD association subsidizing AD research.

The implementation of the proposed strategies would necessarily require additional efforts to increase general public awareness regarding AD pathology and recognition of current failures in research efforts, and ways to prevention. In particular, public initiatives and national campaigns addressing the relevance of nutrition, cognitive training, and physical activity as preventive strategies to reduce the risk of AD and ameliorate AD symptoms, as the ones recently undertaken [62, 63], should be encouraged and supported.

Finally, it is important to implement education and design curricula focused on currently available human-based methods and readouts in both schools and universities, to train new generations of scientists competent in the field of alternatives to animal experimentation, and well versed in the necessity and power of multiscale human-based research approaches.

**Table 3:** List of recommendations to guide new funding strategies

Recommendations		Comments
R1	Implement funding for the production and centralized distribution of AD patient-derived cells (e.g., fibroblasts, peripheral blood cells, iPSCs)	Consider establishing NIH-funded centers to provide investigators with patient-derived cells and already reprogrammed iPSCs. However, this might be proven unnecessary if other entities, such as CIRM and NYSFC, will do this on their own
R2	Allocate funding for research proposals aiming at defining & validating early biomarkers of AD	Current biomarkers measure levels of Aβ (in CSF), and levels of phospho-tau and total tau (in CSF). In this regard, neuroimaging technologies by means of MRI and PET (FDG-PET and amyloid imaging) are particularly suitable to allow early detection of AD and assess therapeutic efficacy *in vivo*. Develop and validate additional portable and non-invasive techniques that can identify predictive biomarkers.
R3	Allocate more funding to research projects focusing on the most prevalent late-onset/sporadic AD	Despite the fact that the majority of AD cases are late-onset, the current number of NIH funded active projects focused on the late-onset/sporadic AD is lower than the number of projects on early-onset and familial AD (81 vs 182, as of July 6th 2015. Data retrieved from http://projectreporter.nih.gov/reporter.cfm)
R4	Allocate funding to centers conducting omics research in human-based settings	This would be relevant considering the need for expensive high throughput technological tools and creation of multidisciplinary teams of experts
R5	Create specific RFAs focused on non-animal/human-based research	One example in this direction to significantly reduce animal experimentation is provided by Europe and UK: for instance, NC3Rs rates projects considering their scientific value as 50% and their contribution to the reduction of animal tests as the remaining 50% of the final score (http://www.nc3rs.org.uk/funding). More directly, a dedicated call should be made for complete and direct alternatives that offer new perspectives and fundamentally ethical approach that do not involve animal experimentation
R6	Increase funding support for basic research studies to speed the discovery process	Recognize the many types and growing applicability of non-animal models in basic research. Dedicated funding should be allocated to high-risk high innovation studies, including the development of non-animal models for research in this area. Not all projects need to be immediately translational in nature
R7	Increase funding to study risk factors and evidence-based prevention approaches to slow the progression of AD	There is an urgent need to increase funding for epidemiological and clinical studies, focused on the impact of specific nutrition, level of physical activity, and level of educational attainment in the onset and progression of AD. Also, increase resources for examining factors across multiple risk and ameliorating variables including: environmental exposure, access to health care, sex and gender, ongoing social and cognitive engagement. Design intervention strategies in large scale cohorts. Dedicate resources to disseminate knowledge of known lifestyle factors to the public at large as well as new incoming information. Randomized clinical trials of individual dietary practices as well as nutritional supplements. Begin with individuals who have low or insufficient nutrient levels and for whom the highest beneficial effects have been observed (Morris, Tangney et al. 2015)
R8	Consider ethno-cultural factors	Epidemiological studies addressing ethnic, cultural variations and implication of lifestyle risk factors would be highly relevant both to smaller communities and lessons that can be extended to the population at large. Collaboration with epidemiological studies in other clinical domains, such as vascular research (Satizabal, Beiser et al. 2016) will be critical for unmasking these complex relationships.

Abbreviations: CIRM, California Institute for Regenerative Medicine; NYSCF, New York Stem Cell Foundation; CSF, cerebrospinal fluid; MRI, magnetic resonance imaging; PET, positron emission tomography; FDG-PET, fluorodeoxyglucose-PET; RFAs, Requests for Applications.
